# Chimeric Virus-like Particle-Based COVID-19 Vaccine Confers Strong Protection against SARS-CoV-2 Viremia in K18-hACE2 Mice

**DOI:** 10.3390/vaccines10050786

**Published:** 2022-05-16

**Authors:** Challika Kaewborisuth, Asawin Wanitchang, Surapong Koonpaew, Kanjana Srisutthisamphan, Janya Saenboonrueng, Rawiwan Im-Erbsin, Manutsanun Inthawong, Piyanate Sunyakumthorn, Theeradej Thaweerattanasinp, Nathiphat Tanwattana, Yuparat Jantraphakorn, Matthew C. Reed, Luis A. Lugo-Roman, Taweewun Hunsawong, Chonticha Klungthong, Anthony R. Jones, Stefan Fernandez, Samaporn Teeravechyan, Eric D. Lombardini, Anan Jongkaewwattana

**Affiliations:** 1 Virology and Cell Technology Research Team, National Center for Genetic Engineering and Biotechnology (BIOTEC), National Science and Technology Development Agency (NSTDA), Pathumthani 12120, Thailand; challika.kae@biotec.or.th (C.K.); asawin.wan@biotec.or.th (A.W.); surapong.koo@biotec.or.th (S.K.); kanjana.sri@biotec.or.th (K.S.); janya@biotec.or.th (J.S.); theeradej.tha@biotec.or.th (T.T.); nathiphat.tan@gmail.com (N.T.); yuparat.jan@gmail.com (Y.J.); samaporn.tee@biotec.or.th (S.T.); 2 Department of Veterinary Medicine, U.S. Army Medical Directorate-Armed Forces Research Institute of Medical Sciences, Bangkok 10400, Thailand; rawiwani.fsn@afrims.org (R.I.-E.); manutsanuni.ca@afrims.org (M.I.); piyanates.fsn@afrims.org (P.S.); matthew.reed.mil@afrims.org (M.C.R.); luis.lugo.mil@afrims.org (L.A.L.-R.); 3 Interdisciplinary Program in Genetic Engineering and Bioinformatics, Graduate School, Kasetsart University, Bangkok 10900, Thailand; 4 Department of Virology, U.S. Army Medical Directorate-Armed Forces Research Institute of Medical Sciences, Bangkok 10400, Thailand; taweewunh.fsn@afrims.org (T.H.); chontichak.fsn@afrims.org (C.K.); anthony.jones.mil@afrims.org (A.R.J.); stefan.fernandez.mil@afrims.org (S.F.); 5 U.S. Army Medical Directorate-Armed Forces Research Institute of Medical Sciences, Bangkok 10400, Thailand; eric.lombardini.mil@afrims.org

**Keywords:** chimeric virus-like particle, vaccine, SARS-CoV-2, COVID-19, K18-hACE2 mice

## Abstract

Virus-like particles (VLPs) are highly immunogenic and versatile subunit vaccines composed of multimeric viral proteins that mimic the whole virus but lack genetic material. Due to the lack of infectivity, VLPs are being developed as safe and effective vaccines against various infectious diseases. In this study, we generated a chimeric VLP-based COVID-19 vaccine stably produced by HEK293T cells. The chimeric VLPs contain the influenza virus A matrix (M1) proteins and the SARS-CoV-2 Wuhan strain spike (S) proteins with a deletion of the polybasic furin cleavage motif and a replacement of the transmembrane and cytoplasmic tail with that of the influenza virus hemagglutinin (HA). These resulting chimeric S-M1 VLPs, displaying S and M1, were observed to be enveloped particles that are heterogeneous in shape and size. The intramuscular vaccination of BALB/c mice in a prime-boost regimen elicited high titers of S-specific IgG and neutralizing antibodies. After immunization and a challenge with SARS-CoV-2 in K18-hACE2 mice, the S-M1 VLP vaccination resulted in a drastic reduction in viremia, as well as a decreased viral load in the lungs and improved survival rates compared to the control mice. Balanced Th1 and Th2 responses of activated S-specific T-cells were observed. Moderate degrees of inflammation and viral RNA in the lungs and brains were observed in the vaccinated group; however, brain lesion scores were less than in the PBS control. Overall, we demonstrate the immunogenicity of a chimeric VLP-based COVID-19 vaccine which confers strong protection against SARS-CoV-2 viremia in mice.

## 1. Introduction

In the two years since the emergence of the severe acute respiratory syndrome coronavirus 2 (SARS-CoV2) and the 2019 coronavirus disease (COVID-19) [1], approximately 64.4% of the world’s population has received at least one dose of a COVID-19 vaccine [2]. Currently, vaccine solutions remain inadequate, with only 14.5% of people in low-income countries having received at least one dose. Furthermore, the successive emergence of the SARS-CoV-2 variants around the globe nullifies the efficacy of most currently available vaccines, which are based on the ancestral virus strain. SARS-CoV-2 is likely to remain the greatest threat to public health worldwide for the foreseeable future and further vaccine development will be necessary to contain this ongoing threat.

Several platforms have been used to develop a vaccine against SARS-CoV-2. These include inactivated viruses, viral vectors (non-replicating viruses), nucleic acids (mRNA/DNA), and proteins [3]. These various types of vaccines have different safety, efficacy, and production profiles. For example, inactivated vaccines are considered extremely safe, but offer a lower efficacy than vaccines based on viral vectors and nucleic acids [4]. On the other hand, while viral vectors elicit robust humoral and cell-mediated immune responses against SARS-CoV-2, pre-existing immunity to their viral components is a drawback that may reduce the vaccination’s effectiveness [5]. In addition, vaccines run the risk of reversion to high pathogenicity, and a biosafety level 3 (BSL3) facility is required to produce SARS-CoV-2-specific vaccines. Given the difficulties in handling live viruses, the requirement for a highly stringent biosafety environment, and the lack of cold chains, protein subunit vaccines are an alternative platform that offers ease of production and safety. Protein-based COVID-19 vaccines have been shown to induce robust neutralizing antibody and T cell responses [6,7], and a number of them have entered clinical trials [8,9,10].

Virus-like particles (VLPs), a type of protein-based vaccine, are self-assembling viral protein structures consisting of multiple subunits that resemble native viruses [11], thus mimicking the immunogenic properties of the natural virus [12]. The size of VLPs is favorable for uptake by antigen-presenting cells leading to the induction of robust innate and adaptive immune responses [13,14,15]. Although the co-administration of adjuvants with VLPs is not necessary due to the multimeric nature of the antigen, the use of adjuvants usually improves their immunogenicity. While VLPs are commonly produced using protein(s) from a single virus type, structural proteins from different viruses can also be used to form chimeric VLPs. This designation for chimeric VLPs is based primarily on the use of heterologous viral proteins that line the inner layer of the cellular plasma membrane to facilitate the budding and egress of the virus. Generally, the enveloped chimeric VLPs have repeating units of immunogenic glycoprotein embedded in the lipid layers and associated with the proteins derived from the different viruses to self-assemble. While several SARS-CoV-2 VLP vaccines based on the envelope (E), matrix (M), and spike (S) proteins have been produced on different expression platforms [16,17,18,19], there are few reports on the development of chimeric SARS-CoV-2 VLP vaccines. One report described chimeric MLV-Gag/S VLPs that could be produced on a large scale in proprietary HEK293 cells and induced strong immune responses and protection in Syrian golden hamsters [20]. In another case, the transient expression of SARS-CoV-S glycoprotein and an influenza matrix (M1) in insect cells was reported, which self-assembled into VLPs capable of inducing neutralizing antibodies and protecting mice from lethal challenges [21]. More recently, the same approach was used to display SARS-CoV-2 S [22]. Although the vaccine could induce neutralizing antibodies in mice, its protective efficacy remains to be evaluated.

In this study, we report the development of a chimeric VLP-based COVID-19 vaccine and its production from a HEK293T cell line stably expressing SARS-CoV-2 spike fused with an influenza A transmembrane and the cytoplasmic tail of HA and the M1 protein. The intramuscular administration of a prime-boost regimen to mice demonstrated the strong induction of the humoral response, resulting in antibodies with neutralizing activity against various variants of concern as well as the strong systemic suppression of SARS-CoV-2 in the blood. Our studies here demonstrate that SARS-CoV-2-based VLPs can be developed as a potentially safe and effective vaccine against the SARS-CoV-2 infection, although additional refinements of the vaccine, especially its formulation or dosage, are required to achieve desirable results.

## 2. Materials and Methods

### 2.1. Cell Lines

Human embryonic kidney (HEK) 293T cells were purchased from ATCC^®^ (CRL-3216); ATCC^®^, Manassas, VA, USA). Cells were maintained in Opti-MEM^TM^ I reduced serum media (Thermofisher Scientific, Waltham, MA, USA) supplemented with 10% fetal bovine serum (FBS) (Thermofisher Scientific, Waltham, MA, USA). Vero E6 cells were cultured in EMEM containing 2% FBS, 1% L-glutamine, 1% penicillin-streptomycin (Thermofisher Scientific), 40 µg/mL gentamicin (Thermofisher Scientific), and 0.25 µg/mL fungizone (Thermofisher Scientific). All cells were maintained in a 5% CO_2_ incubator at 37 °C.

### 2.2. Plasmid Construction

The codon-optimized spike construct comprising of SARS-CoV-2 spike (Wuhan-Hu-1 (EPI_ISL_402125)) with the disruption of the furin cleavage site and deletions of the transmembrane and cytoplasmic domains, fused to the transmembrane and cytoplasmic tail of the HA protein from influenza A/Indonesia/5/2005 (H5N1) [21] (SΔfurin-HAcyt), was synthesized and cloned into the pUbEm plasmid containing the Emerald GFP reporter gene, so-called pUbEm-S. The codon-optimized M gene of influenza A/Indonesia/5/2005 (H5N1) [21] was synthesized and cloned into the pUB-mCherry plasmid, so-called pUbmC-M1.

### 2.3. Establishment of a Stable Cell Line Producing Chimeric Virus-like Particles

To generate lentiviruses encoding S (Lenti-GFP-S) or M1 (Lenti-mCherry-M1) for cell transduction, HEK293T cells were seeded in a 6-well plate and transfected with 1.5 µg pUbEm-S or pUbmC-M1, 1.0 µg pCMVΔ8.91, and 0.5 µg pMD.G using Fugene HD (Promega, Madison, WI, USA) at a 3:1 ratio. Supernatants containing Lenti-mCherry-M1 or Lenti-GFP-S were harvested 3 days post-transfection and clarified by centrifugation.

To establish a stable cell line capable of continuously producing chimeric S-M1 virus-like particles (S-M1 VLPs), HEK293T cells seeded in a 6-well plate were transduced with Lenti-mCherry-M1 in the presence of 10 µg/mL polybrene. After 24 h, the media was replaced. Seventy-two hours post-transduction, cells were sorted for high fluorescence by flow-activated cell sorting (FACS) using the FACSAria^TM^ Fusion flow cytometer (BD Biosciences, CA, USA). The M1 positive cells were expanded, seeded in a 6-well plate, and further transduced with Lenti-GFP-S. The resulting cells expressing S and M1 were selected for green fluorescence of mEmerald GFP protein by FACS.

### 2.4. Expression and Purification of S-M1 VLPs

HEK293T cells expressing S-M1 VLPs (HEK293T-S-M1) were grown in Opti-MEM^TM^ I reduced serum media (Thermofisher Scientific) in tissue-treated 15-cm^2^ dishes (5 dishes per production) for 72 h. The cell supernatant (up to 100 mL) was collected and concentrated through a 50 kDa Amicon^®^ Ultra Centrifugal Filter Unit (Merck, Darmstadt, Germany) (final volume of 25 mL). The concentrated sample was subjected to ultracentrifugation through a 20% glycerol cushion at 135,000× *g*, 4 °C for 4 h. Cell pellet containing S-M1 VLPs was harvested and re-suspended with 500 µL of phosphate buffer saline (PBS). The S-M1 VLPs were quantified by dot-blot analysis against rabbit α-spike antibodies. The purified spike protein expressed in HEK293T cells was used as a standard.

### 2.5. Immunofluorescence Assay

HEK293T cells expressing S-M1 VLPs were grown on coverslips in 6-well plates for 24 h. The cells were fixed with 4% paraformaldehyde at 4 °C for 20 min, blocked, and permeabilized with 3% bovine serum albumin (BSA) and 0.3% Triton-X for 30 min. The cells were then stained with mouse anti-S (Sino Biological, Beijing, China) and rabbit anti-influenza A M1 (Bio-Rad, Hercules, CA, USA) as primary antibodies for 1 h at room temperature. After incubation, cells were washed three times with PBST and then stained with goat α-rabbit Alexa Fluor^®^ 488 (Abcam, Cambridge, UK) and goat α-mouse IgG Alexa Fluor^®^ 647 (Abcam). After a wash step, cells were mounted with Prolong^TM^ Gold Antifade Mountant with DAPI (Invitrogen, Carlsbad, CA, USA). Samples were visualized using Fluoview^TM^ FV1000 confocal microscopy (Olympus, Tokyo, Japan).

### 2.6. Western Blot Analysis

The presence of S-M1 VLPs in stably expressed S-M1 cells and purified S-M1 VLPs was assessed by Western blot analysis. Cells were lysed using the Pierce™ mammalian cell lysis buffer (Thermofisher Scientific). Protein samples were loaded onto polyacrylamide gels and transferred to nitrocellulose membranes. Membranes were probed with rabbit α-S (Sino Biological) and α-influenza A M1 (Bio-Rad). Goat α-rabbit IgG-HRP (KPL, Milford, MA, USA) and α-mouse IgG-HRP antibodies were used as secondary antibodies for chemiluminescence detection with the ChemiDoc™ XRS+ imager (Bio-Rad).

### 2.7. Transmission Electron Microscopy

Five microliters of the S-M1 VLPs in PBS were negatively stained with 3% phosphotungstic acid solution (PTA) for 30 sec. The S-M1 VLPs/PTA mixture was incubated on a carbon-coated copper grid (EMS, PA, USA) for 1 min. For immunogold labeling, the S-M1 VLPs/PTA mixture was adsorbed onto a carbon-coated copper grid for 30 min and then blocked with 1% bovine serum albumin (BSA) for 1 h. The grids were then incubated with rabbit α-spike antibody (Sino Biological) at a dilution of 1:30 in PBS for 1 h and rinsed six times with PBS, followed by incubation with goat α-rabbit IgG antibodies (conjugated with 10-nm gold particles) at a dilution of 1:100 in PBS for 1 h. After washing, the samples were fixed in 2% glutaraldehyde for 5 min and negatively stained with 3% PTA. The grid was kept in a vacuum incubator before observation with a transmission electron microscope (Hitachi H7700, Tokyo, Japan) at 80 kV.

### 2.8. Immunization in BALB/c Mice

To determine the immunogenicity of the vaccine in a mouse model, 8-week-old female BALB/c mice were purchased from Nomura Siam International Co., Ltd. (Bangkok, Thailand). Mice were divided into 4 groups (*n* = 5/group) and received the vaccine using a prime-boost regimen (3-week intervals). Mice were injected intramuscularly with the S-M1 VLPs with or without alum adjuvant. At 3 weeks after boosting, mice were euthanized for collection of serum and spleen samples. Sera were subjected to ELISA to determine IgG levels. Spleens were processed for splenocyte isolation and subjected to a mouse IFN gamma ELISpot assay (CTL, Shaker Heights, OH, USA) according to the manufacturer’s protocol.

### 2.9. Quantification of Specific Anti-SARS-CoV-2 Spike IgG by ELISA

SARS-CoV-2 S RBD-specific antibody responses were determined using an in-house anti-IgA/IgG SARS-CoV-2 S RBD ELISA. Briefly, 96-well high-binding ELISA plates were coated with 0.1 µg of SARS-CoV-2 S RBD per well and incubated overnight at 4 °C. Coated plates were washed six times (6×) with phosphate buffer saline containing 0.1% Tween 20 (PBST) before blocking with 5% skim milk in PBST (blocking solution) and then incubated at room temperature (RT) for 1 h. Sera were two-fold diluted with blocking solution before addition of 100 µL into duplicate wells. After incubation for 1 h at RT, plates were washed (6×) with PBST before adding 100 µL of HRP-conjugated goat α-human IgA (1:10,000) or HRP-conjugated goat α-human IgG (1:40,000), and incubating the plates at RT for 1 h. Plates were washed (6×) with PBST before adding 100 µL of TMB substrate and incubating at RT for 15–20 min. Enzyme activity was stopped by adding 50 µL of 2 M sulfuric acid and OD values at 450 nm were immediately measured using a plate reader. Values equal or higher than the cut-off value (≥3-fold of the mean OD_450_ of the negative control) were considered positive for IgA or IgG antibodies. Using this criterion, the IgA assay performance had 85.7% sensitivity and 97.3% specificity while the IgG assay exhibited 96.0% sensitivity and 96.7% specificity.

### 2.10. SARS-CoV-2 Microneutralization

A CPE-based microneutralization assay (MN) with colorimetric readout was used to determine neutralizing (NT) antibodies against live SARS-CoV-2 Wuhan-Hu-1 strain virus. Cell (CC) and virus control (VC) wells were included. Sera were heat-inactivated at 56 °C for 30 min and two-fold serially diluted starting from 1:10. Sera were mixed with 100 TCID50 of SARS-CoV-2 virus and incubated for 1 h at 37 °C in a 5% CO_2_ incubator. Then, the serum–virus mixtures were inoculated in duplicate onto Vero E6 cells seeded in 96-well plates and incubated for 3 days at 37 °C, and 5% CO_2_. Cells were fixed with methanol/acetone and blocked with 1X PBS containing 2% bovine serum albumin (BSA) and 0.05% Tween 20. Cells were then stained with SARS-CoV/SARS-CoV-2 nucleocapsid mAb (Sino Biological) as the primary antibody and HRP-conjugated goat α-rabbit polyclonal antibody (Dako, Denmark A/S). After the washing steps, TMB substrate was added (KPL) into each well and the reaction was stopped with 1N HCl. Absorbance was measured at 450 nm by an ELISA microplate reader. The percentage of virus infectivity in VC and samples was calculated based on the OD of CC using the formula infectivity (%) = (OD of CC − OD of the sample) × 100. The MN50 titer was calculated by determining the half-maximal inhibitory dilution. All procedures were performed in a BSL-3 laboratory following a standard neutralization assay.

### 2.11. SARS-CoV-2 Pseudotyped Virus Neutralization Assay

Lentiviral vectors carrying the firefly luciferase reporter were pseudotyped with the full-length S of various SARS-CoV-2 variants of concern (VOC), namely alpha, beta, gamma, delta, and omicron, using methods as described previously [23,24]. Sera were heat-inactivated at 56 °C for 30 min, incubated with 1 × 10^4^ relative light units (RLU)/mL of the pseudotyped virus at 37 °C for 1 h, then diluted (two-fold serial dilutions) in DMEM supplemented with 10% FBS. The mixtures were added to individual wells of opaque, white 96-well microplates. HEK 293T cells expressing ACE2 and TMPRSS2 cells (1 × 10^4^ cells) were suspended in 50 µL DMEM supplemented with 10% FBS, and then added to each well. Plates were incubated at 37 °C, 5% CO_2_ for 48 h, and a readout was performed by removing supernatants and adding 25 µL of Bright-Glo^TM^ luciferase substrate (Promega) to each well. Entry of pseudotyped viruses, as quantified by firefly luciferase signal, was measured using the Synergy HTX plate reader (BioTek, Winusky, VT, USA). Values were normalized against signals from no-serum controls. The PVNT50 value was calculated by determining the half-maximal inhibitory dilution.

### 2.12. SARS-CoV-2 Challenge Test in K18-hACE2 Mice and Sample Collection

Female K18-hACE2 mice (6 weeks old) strain 2B6.Cg-Tg (K18-ACE2)2Prlmn/J were purchased from InVivos (Singapore) and maintained in micro-isolator cages in an ABSL-2 facility before the SARS-CoV-2 challenge or in an ABSL-3 facility after the challenge. Mice had nesting and shelter facilities as part of their cage environment and ad libitum access to commercial pelleted diets and chlorinated water.

Twelve mice were included and randomly divided into two groups (*n* = 6). Mice were either intramuscularly (IM) administered S-M1 VLPs or PBS in a three-week interval prime-boost schedule. Three weeks following the second immunization (day 42), all mice were intranasally (IN) challenged with 2 × 10^4^ PFU/ 50 μL of the SARS-CoV-2 Wuhan reference strain hCoV-19/Hong Kong/VM20001061/2020. Observations for clinical signs of disease including body weight, inappetence, and behavioral anomalies were performed daily post-infection. Animals were humanely euthanized by CO_2_ inhalation upon meeting euthanasia criteria or at pre-determined endpoints, following institutional and AVMA guidelines (2020 edition).

At the study endpoint, animals were euthanized. Serum samples were analyzed for viremia (qRT-PCR) and SARS-CoV-2-specific antibody responses (qualitative IgG & IgA spike-RBD). Major organs were collected for viral RNA determination, histopathology, and SARS-CoV-2-specific-in situ hybridization. SARS-CoV-2-specific cellular immune responses were determined at the study endpoint in splenocytes by the ELISpot assay and supernatants of stimulated cells were used for cytokine profiling.

### 2.13. Virus Titration

Titers of viable virus in tracheal lavage specimens were determined using a 50% tissue culture infectious dose assay (TCID_50_). All procedures were performed in a BSL-3 laboratory following a standard neutralization assay. Tracheal lavage samples were serially diluted 5-fold (starting from undiluted to 10^−5^) with Vero E6 culture media, before inoculation onto Vero E6 cells in 96-well plates. Cell control (CC) wells were included in every single plate. After incubation for 1 h, the infectious medium was removed before replenishing with Vero E6 culture media and cultured for 5 days at 37 °C in a 5% CO_2_ incubator. Cells were stained with 0.02% neutral red in PBS, pH 7.2 ± 0.1, and incubated for 1 h at room temperature (RT). Then, cells were washed twice with 1× PBS before adding lysis solution (50% absolute ethanol and 1% acetic acid in distilled water). After incubation for 15 min at RT, absorbance (OD) values at 540 nm were determined using the SpectraMax microplate reader (Molecular Devices, CA, USA). The cut-off value was calculated by dividing the mean OD of CC wells by two. Any test well with OD values less than the cut-off was scored as positive for viral growth. TCID_50_ value for each sample was calculated using the Reed–Muench method [25].

### 2.14. Viral RNA Quantification by Real-Time RT-PCR

Viral RNA in serum (140 µL) and tissue (20–30 mg) samples was extracted using the QIAamp viral RNA mini kit (QIAGEN, Hilden, Germany) or the RNeasy Mini kit (QIAGEN) according to the manufacturer’s instructions. A total volume of 50 µL of viral RNA was obtained for each sample. Five microliters of each RNA sample were used for RT-qPCR targeting the N1 region of the SARS-CoV-2 nucleocapsid (N) gene, using the US-CDC procedure [26] and in vitro SARS-CoV-2 RNA transcripts (IVTs) as standards. The RT-qPCR was performed using the SuperScript III Platinum One-Step Quantitative RT-PCR System (Invitrogen) with the US-CDC 2019-nCoV-N1 combined primer and probe mix. A one-step RT-PCR consisting of a 30-min RT step at 50 °C, 5 min of Taq polymerase inhibitor inactivation at 95 °C, followed by 45 at 95 °C for 15 s and 58 °C for 30 s was performed on an ABI 7500 Fast Real-Time PCR system (Applied Biosystems, MA, USA). Three internal controls included a no-template control (NTC), a negative extraction control (NEC), and a positive extraction control (PEC). Serial dilutions of IVT RNA standards were run along with test samples in each experiment. The number of copies of viral RNA per sample was derived from standard curves generated from serial dilutions of IVTs (5, 50, 5 × 10^2^, 5 × 10^3^, 5 × 10^4^, and 5 × 10^5^ RNA copies or genomic equivalent (GE) per reaction). The GE per ml of virus in a serum sample was calculated using the formula: the number of copies per reaction × 10,000 × the volume of a serum sample used (µL) for extraction. The GE per gram of virus in a tissue sample was calculated using the formula: number of copies per reaction × 10,000 × weight of tissue sample (mg) used for extraction.

### 2.15. Splenocyte Isolation

Spleens were collected from euthanized mice and placed in 50-mL conical tubes containing 5 mL of RPMI media with 10% heat-inactivated FBS supplemented with L-glutamine and penicillin-streptomycin antibiotics to prepare single-cell suspensions. Each spleen was placed on a Petri dish, minced into small pieces using a surgical blade, and then mashed against the surface of a 70-µm cell strainer over a 50-mL conical tube. Red blood cell contaminants were removed by incubation with Gibco ACK lysing buffer (Thermofisher Scientific) for 5 min at room temperature. Cell suspensions were washed by centrifugation at 300× *g* for 5 min and resuspended in RPMI containing 10% FBS for counting before adjusting to the desired cell quantity. All procedures were performed in a BSL-3 laboratory.

### 2.16. IFN-γ ELISpot Assay

To assess SARS-CoV-2-specific cellular immune responses in splenocytes, an ex vivo IFN-γ ELISpot assay was performed using an ELISpot Mouse IFN-γ ELISpot PLUS kit (ALP) (Mabtech, Sweden) in a BSL-3 laboratory according to the manufacturer’s protocol. Briefly, 96-well-PVDF membrane plates pre-coated with monoclonal antibody clone AN18 were washed and then blocked with RPMI media containing 10% FBS, supplemented with L-glutamine and penicillin-streptavidin antibiotics for at least 30 min at 37 °C. The media was discarded and 8 × 10^5^ splenocytes isolated from the spleen were stimulated with 2 µg/mL PepMix™ SARS-CoV-2 S peptide pools based on the Wuhan-Hu-1 strain (JPT Peptide Technologies, Berlin, Germany) for 18–20 h at 37 °C in a 5% CO_2_ incubator. Phytohemagglutinin (PHA) at a final concentration of 10 µg/mL was used as a positive control and media alone was used as a negative control. After the incubation period was complete, culture supernatants were collected for cytokine profiling and IFN-γ-secreting cells were detected by incubation of biotinylated detection antibody (clone R4-6A2) at a 1:1000 dilution for 2 h at room temperature. Then, the plates were washed and further incubated with streptavidin-conjugated alkaline phosphatase at a 1:1000 dilution for 1 h at room temperature. After further washing, BCIP/NBT-plus substrate was added. Spot numbers were counted by an automated ELISpot reader with Smart Count™ ImmunoSpot 6 software (S6 Universal FluoroSpot, Cellular Technology Limited, Shaker Heights, OH, USA). Results were reported as spot-forming cells (SFC) per one million cells. The non-specific background was subtracted from the experimental readings.

### 2.17. Cytokine Profiling Bio-Plex Assay

Culture supernatants were collected from the ELISpot assay plate and inactivated with Triton-X 100 at a final concentration of 0.5% for 30 min at room temperature. Cytokine profiling was performed using the Bio-Plex Pro Mouse Cytokine 23-plex Assay (Bio-Rad) based on magnetic beads, and the final reaction was detected using the Bio-Plex 200 analyzer. The 23 cytokines consisted of IL-1α, IL-1β, IL-2, IL-3, IL-4, IL-5, IL-6, IL-9, IL-10, IL-12 (p40), IL-12 (p70), IL-13, IL-17A, eotaxin, G-CSF, GM-CSF, IFN-γ, KC, MCP-1 (MCAF), MIP-1α, MIP-1β, RANTES, and TNF.

### 2.18. Histopathology

Necropsies of mice were performed in an ABSL3 laboratory. Major organs harvested postmortem for histopathologic analysis included lung, lymphoid tissue (lymph nodes, spleen, and thymus), brain, nasal turbinates, and adrenal glands. All tissues were immediately fixed in 10% neutral buffered formalin for at least 48 h, then paraffin-embedded, and sectioned at 4 µm thickness. Embedded tissue sections were then de-paraffinized, and rehydrated through a series of xylene substitutes and isopropanol. Tissue sections were stained with hematoxylin-eosin (H&E) and analyzed by a board-certified veterinary pathologist.

### 2.19. In-Situ Hybridization for SARS-CoV-2 RNA

To detect the presence of SARS-CoV-2 RNA in mouse tissue samples, formalin-fixed paraffin-embedded (FFPE) lung and brain tissues were examined using the RNAscope^®^ in situ hybridization (ISH) assay. The V-nCoV2019-S RNAscope^®^ probe (Advanced Cell Diagnostics, Newark, CA, USA), specific for the SARS-CoV-2 S gene based on isolate Wuhan-Hu-1 (Genbank accession number NC_045512.2, targeting region 21631-23303), was used. The RNAscope^®^ ISH assay was performed using an RNAscope 2.5 HD Red Detection Kit (Advanced Cell Diagnostics) as follows. FFPE tissue slides were de-paraffinized and treated with hydrogen peroxide for 10 min at room temperature, followed by target retrieval in 1× target retrieval solution in a steamer at least 99 °C for 15 min. Slides were then incubated with Protease Plus (Advanced Cell Diagnostics) for 30 min at 40 °C in a HybEZ™ oven (Advanced Cell Diagnostics) and subsequently incubated with the SARS-CoV-2 specific probe for 2 h at 40 °C in the HybEZ™ oven. The signal was amplified using a specific set of amplifiers (AMP1-6), as recommended by the manufacturer, and was detected using a Fast Red solution for 5 min at room temperature. Slides were counterstained with 50% Gill hematoxylin III (Sigma Aldrich, MO, USA) for 2 min and extensively washed with tap water. Next, the slides were dehydrated in a 60 °C drying oven until completely dry and then immersed in xylene before mounting with mounting medium.

### 2.20. Statistical Analyses

Differences in means between groups were analyzed by the one-way method ANOVA using GraphPad Prism 5.0 (GraphPad Software Inc., San Diego, CA, USA). *p* values of <0.05 were considered statistically significant. All data are presented as means ± standard error of means (SEM), unless otherwise stated.

### 2.21. Ethics Statement

Immunogenicity testing in BALB/c mice was conducted following the guidelines of the Institutional Animal Care and Use Committee (IACUC) of the National Center for Genetic Engineering and Biotechnology, Thailand (BT-Animal 22/2563). The SARS-CoV-2 challenge study in K18-hACE2 mice was performed under an approved Institutional Animal Care and Use Committee (IACUC) protocol at the Armed Forces Research Institute of Medical Sciences (AFRIMS), Thailand, and AAALAC International-accredited facility. The IACUC protocol number was PN21-03. The animal research was conducted in compliance with Thai laws, the Animals for Scientific Purposes Act, B.E. 2558 (A.D. 2015), the Animal Welfare Act, and all applicable U.S. Department of Agriculture, Office of Laboratory Animal Welfare, and U.S. Department of Defense guidelines. All animal research adhered to the Guide for the Care and Use of Laboratory Animals, NRC Publication (8th Edition) [27].

## 3. Results

### 3.1. Design and Characterization of Chimeric VLPs Containing S and M1 Proteins (S-M1 VLPs)

**Figure 1:**
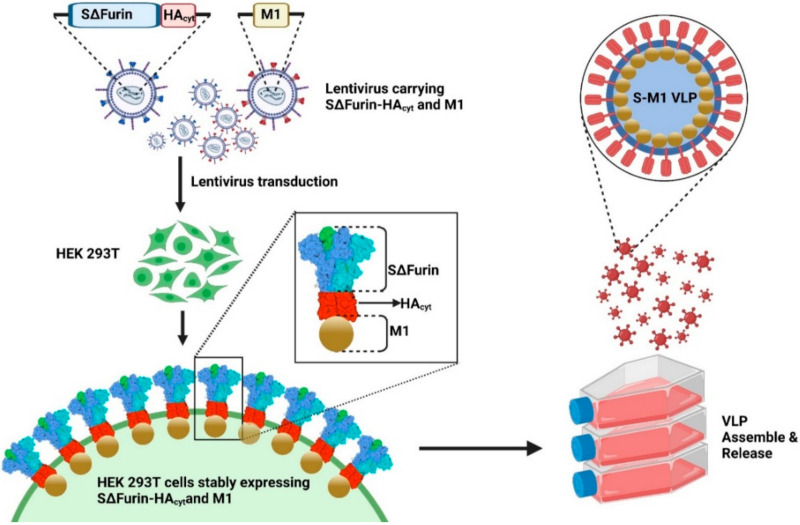
Development of a cell line for the production of chimeric SARS-CoV-2 VLP. Lentiviruses carrying either a SARS-CoV-2 S gene with a deletion of the RRAR motif (ΔFurin) and fused to an influenza virus HA transmembrane and cytoplasmic tail (HA_cyt_), or an influenza A M1 gene were used to transduce HEK293T cells. The resulting HEK293T cells express S on the cell surface, and associate with M1 proteins. The M1 proteins induce the cell membrane budding, resulting in the release of S-M1 VLP into the cell culture supernatant while the cell line is maintained in culture.

Influenza virus-based VLPs were generated based on a chimeric SARS-CoV-2 spike (S) design. A codon-optimized S sequence based on the Wuhan-Hu-1 S ectodomain was synthesized with a deletion in the polybasic (RRAR) motif of the furin cleavage site and fused to the transmembrane and cytoplasmic tail of an influenza A/Indonesia/5/2005 (H5N1) hemagglutinin (HA) (Figure 1) to promote an interaction with the matrix (M1) protein. To facilitate production, we opted to generate a human embryonic kidney (HEK) 293T cell line stably expressing the chimeric S construct together with the matrix (M1) gene. Budding induced by the M1 protein would lead to the incorporation of the chimeric S due to the M1 interaction with the HA cytoplasmic domain. This approach would allow cells to continuously release chimeric S-M1 VLPs into the cell culture supernatant, eliminating the need for the transient transfection of plasmids.

**Figure 2:**
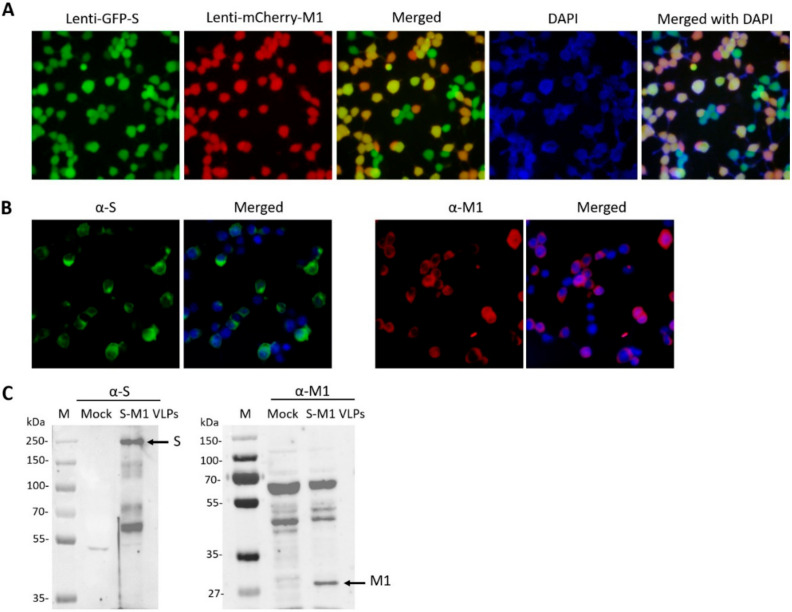
Characterization of the HEK293T-S-M1 cell line. (**A**) HEK293T cells were transduced with Lenti-GFP-S and Lenti-mCherry-M1. Cells were treated with DAPI before fluorescence microscopy. (**B**) HEK293T-S-M1 cells were seeded into an 8-well chamber slide. After fixation and blocking, cells were stained with either rabbit α-S antibodies followed by goat α-rabbit Alexa Fluor^®^ 488, or α-M1 antibodies followed by goat α-mouse IgG Alexa Fluor^®^ 647. Slides were imaged by fluorescence microscopy. (**C**) HEK293T-S-M1 cell lysates were harvested and lysed for Western blotting. Lysates were either probed with rabbit α-S or mouse α-M antibodies. Goat α-rabbit or α-mouse IgG HRP were used as secondary antibodies.

We first cloned the M1 gene into a lentiviral vector encoding an mCherry reporter gene, resulting in Lenti-mCherry-M1, and then the chimeric S construct separately into a second lentiviral vector encoding a GFP reporter gene, resulting in Lenti-GFP-S. HEK293T cells were first transduced with Lenti-mCherry-M1, selected for red fluorescence, and then transduced with Lenti-GFP-S before being selected for green fluorescence. This resulted in cells with dual red and green fluorescence (Figure 2A). The staining of the cells with an anti-S or M1 antibody revealed the expression of S and M1 in these cells (Figure 2B). Cells were harvested and lysed for Western blotting. Probing with anti-S and anti-M1 antibodies confirmed the expression of uncleaved S and M1 in these cells (Figure 2C).

**Figure 3:**
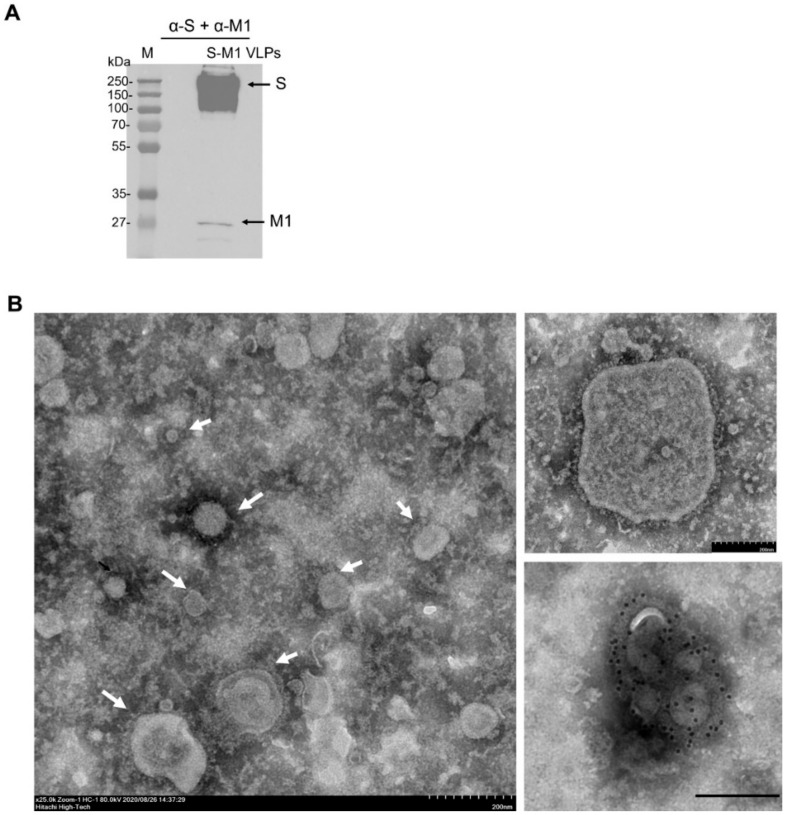
Characterization of S-M1 VLPs. (**A**) Purified VLPs were lysed and analyzed by Western blotting using rabbit α-S or mouse α-M antibodies. Goat α-rabbit or α-mouse IgG HRP were used as secondary antibodies. (**B**) Purified VLPs were negatively stained with the phosphotungstic acid solution. Particles were incubated with rabbit α-S antibodies followed by gold-conjugated α-rabbit antibodies for immunogold labeling. Particles were visualized by TEM. Scale bars are 200 nm.

To confirm the release of S-M1 VLPs into the cell culture supernatant, supernatants were harvested and concentrated using a 50-kDa Amicon centrifugal filter before purification by ultracentrifugation. Western blotting of the concentrated particles revealed a full-length expression of S and M1 (Figure 3A). The S-M1 VLPs quantified by the Bradford protein assay were compared by dot blotting with the purified, mammalian-cell-derived Wuhan-Hu-1 S ectodomain [28], using decreasing amounts of the total protein. Finally, VLPs were imaged using TEM. This revealed pleiomorphic particles of a heterogeneous size with the characteristic crown of S proteins covering the lipid surface (upper right corner). The size of the VLPs ranged from 80 to 200 nm. Immunogold staining of S using a polyclonal rabbit α-S antibody targeting the receptor-binding domain (RBD) revealed the presence of S on particle surfaces (lower right inset) (Figure 3B). These results demonstrate the generation of an HEK293T cell line stably producing VLPs, incorporating our chimeric S on the particle surface.

### 3.2. S-M1 VLPs Elicit Neutralizing Antibodies in BALB/c Mice

**Figure 4:**
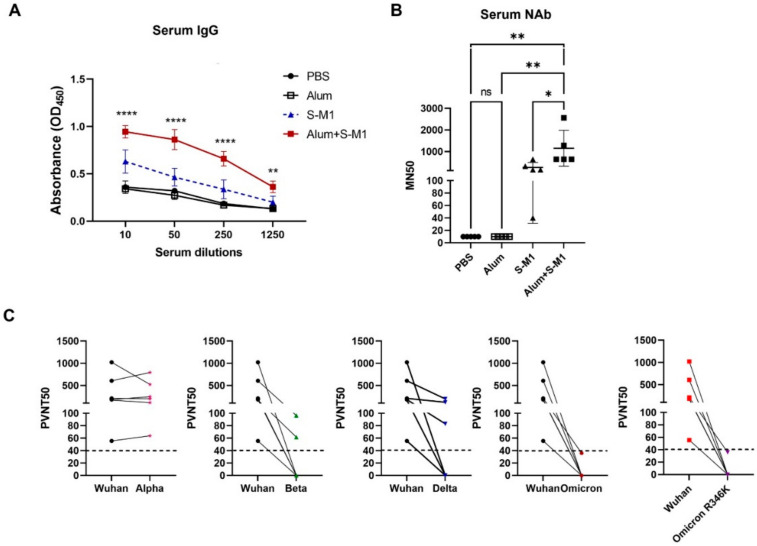
Characterization of neutralizing antibodies in vaccinated BALB/c mice. (**A**) S proteins coated on ELISA plates were incubated with two-fold serial diluted sera from individual mice. Serum IgG was detected with anti-mouse IgG antibodies and visualized by addition of HRP substrate. IgG titers were compared and represented the statistical differences between S-M1 and Alum+S-M1 groups. (**B**) Vero E6 cells seeded in 96-well plates were incubated with live SARS-CoV-2 virus premixed with sera from individual mice. SARS-CoV-2 infection was detected with rabbit α-SARS-CoV-2 N antibodies and visualized by addition of HRP substrate. (**C**) HEK293T-hACE2-TMPRSS2 cells were incubated with S-pseudotyped reporter lentiviruses premixed with increasing dilutions of sera from individual mice that had received alum-S-M1 VLPs. Neutralization was quantified by measuring luciferase activity. Error bars represent means ± sem. ns, no statistical difference; * *p* < 0.05, ** *p* < 0.01, **** *p* < 0.0001.

To evaluate the immunogenicity of our S-M1 VLPs, 5 µg of purified VLPs (25 µL) mixed with 25 µL of alum or PBS were injected intramuscularly into female BALB/c mice on days 0 and 21. Sera were collected on day 42 for the analysis of antibody responses (Figure 4). The sera showed a significant induction of S-specific IgG and neutralizing antibodies, with a more efficient induction when VLP was mixed with alum (Figure 4A,B). Using a pseudotyped virus-based neutralization assay, we confirmed the potent neutralizing activity of sera from mice inoculated with VLP-alum. Using pseudovirus-based neutralization assays, we compared the neutralizing activity of the Wuhan-Hu-1 strain against the variants of concern (Figure 4C). Against the alpha variant, all serum samples showed equal or increased neutralization. For all other variants, individual sera generally exhibited decreased neutralization, particularly against the omicron variants.

### 3.3. S-M1 VLP Vaccination Prevents Viremia and Confers Partial Protection in Immunized K-18-hACE2 Mice

**Figure 5:**
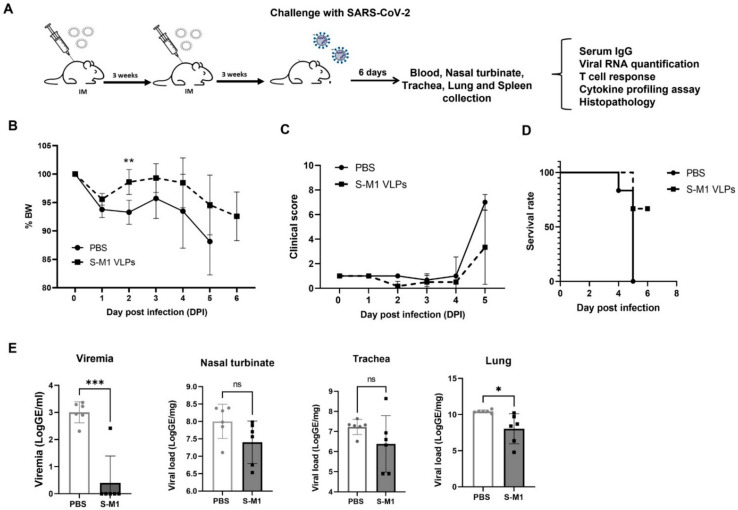
Efficacy of VLP vaccine in K-18 mice. (**A**) K-18 mice were immunized on days 0 and 21 with either PBS or S-M1 VLP mixed with alum. Three weeks after boosting, mice were challenged with live SARS-CoV-2 virus. After sacrifice, various organs were collected for further testing. (**B**,**C**) After the challenge with live SARS-CoV-2 virus, mice were examined daily for (**B**) weight changes and (**C**) physical and behavioral signs of disease, which were scored on a scale from 0 to 10. 0 indicates no indication of disease. (**D**) Survival rates for calculated for each test group over the 6 days before sacrifice. Mice meeting humane endpoints were sacrificed before the experimental endpoint. (**E**) Serum samples and nasal turbinate, trachea, and lung tissue samples were collected and assessed for viral loads in individual mice by RT-qPCR. Error bars represent means ± sem. ns, no statistical difference; * *p* < 0.05, ** *p* < 0.01, *** *p* < 0.001.

To test whether the high neutralizing activity observed in the immunogenicity assays also affects protection against the SARS-CoV-2 infection, we performed immunogenicity and efficacy testing in K18-hACE2 mice, which are transgenic C57BL/6J mice expressing human ACE2 on various epithelial surfaces, including the respiratory tract [29]. The mice were injected intramuscularly with PBS or 5 µg of purified VLP mixed with 25 µL of alum, with a three-week interval between the prime and boost (Figure 5A). No serious adverse effects were observed in either the control or vaccinated groups. A qualitative analysis of the SARS-CoV-2 S receptor-binding domain (RBD)-specific IgG antibody responses was performed. The results showed that 83.3% of mice (5 of 6) seroconverted within 14 days after the first dose. All of the mice seroconverted after the second dose. SARS-CoV-2 RBD-specific IgG was undetectable in all controls after the two doses of mock vaccination.

Three weeks after boosting, the mice were challenged with the ancestral strain of SARS-CoV-2. Following the challenge, none of the animals exhibited any clinical symptoms or weight loss during the first 3 days (Table 1, Figure 5B). A minority of animals in the control group showed mild clinical symptoms with lethargy and ocular discharge at 4 days post-infection (dpi). The onset of symptoms was abrupt and manifested by 5 dpi in the control groups (6 of 6), with clinical scores ranging from 3 to 10 (maximum score = 10; Figure 5C). All mice in the PBS control group eventually exhibited severe clinical signs and met criteria for early euthanasia at 5 dpi (Figure 5D). The mice in the S-M1 VLP group exhibited moderate clinical signs and respiratory symptoms after 5 dpi, but most (3/6) did not meet the criteria for early euthanasia (Figure 5B–D).

Mice were analyzed for the viral load in the blood and respiratory tract by RT-qPCR (Figure 5E). Viremia was undetectable in most of the vaccinated animals (5 of 6) compared to 10^3^ genomic equivalents (GE)/mL in the control animals. A trend toward a lower viral load was observed in the nasal turbinates and trachea, but the differences did not reach statistical significance. However, the S-M1 VLP vaccination significantly reduced the viral load in the lungs. On the other hand, the VLP vaccination was unable to elicit strong protective effects in the respiratory tract, indicating that this approach likely did not elicit much mucosal immunity. Nevertheless, coupled with clinical sign observations, these results suggested that the S-M1 VLP vaccination might be effective in preventing the systemic spread of SARS-CoV-2 from the lungs, limiting the disease to the respiratory system.

### 3.4. S-M1 VLPs Induces T Cell Responses after SARS-CoV-2 Challenge in K18-hACE2 Mice

**Figure 6:**
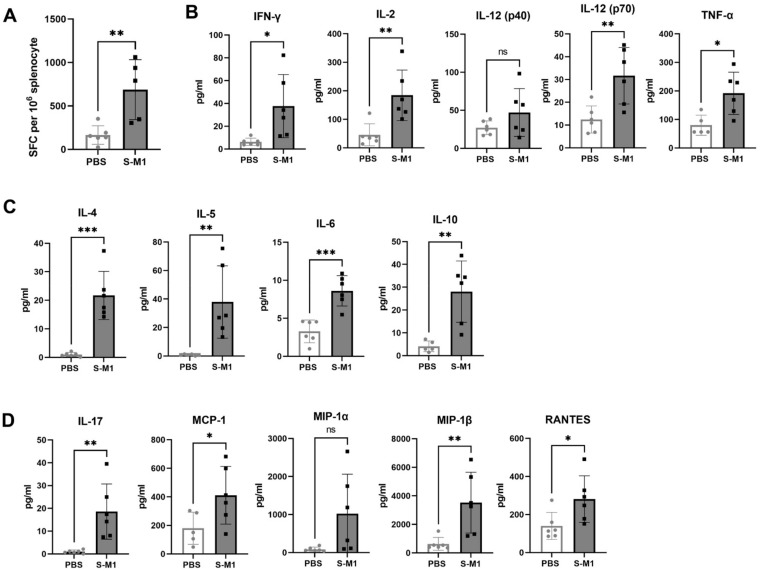
Cellular immune responses to S-M1 VLP vaccination. Splenocytes were isolated from mouse spleens and stimulated with a SARS-CoV-2 peptide pool. (**A**) An ELISpot assay was performed to detect IFN-γ-secreting cells and spots were counted using an automated ELISpot reader. (**B**–**D**) Supernatants from stimulated splenocytes were analyzed for cytokine expression using a magnetic bead-based multiplex assay. Error bars represent means ± sem. ns, no statistical difference; * *p* < 0.05, ** *p* < 0.01, *** *p* < 0.001.

Cellular immune responses were assessed by the quantification of S-specific T cells in the spleen (Figure 6A) using ELISpot. A significantly increased number of S-specific T cells was observed in the vaccinated group compared to the control group. This systemic response may have been the reason for the significant effect of the VLP vaccination on viremia. Cytokines released by splenocytes pulsed with overlapping peptides covering the entire length of the SARS-CoV-2 S protein were measured. Figure 6B,C show the elevated Th1 (Figure 6B) and Th2 cytokine levels (Figure 6C) and the increased levels of the chemokines involved in T-cell and monocyte chemotaxis (Figure 6D) compared to those of control mice. These results suggest that S-M1 VLP triggered a balanced Th1/Th2 response. Baseline concentrations of Th1/2 cells and inflammatory cytokines were also detected in splenocytes obtained from SARS-CoV-2-challenged control mice, possibly as part of the innate immune mechanism in response to the primary viral infection.

### 3.5. S-M1 VLPs Do Not Substantially Lessen Disease Severity

**Figure 7:**
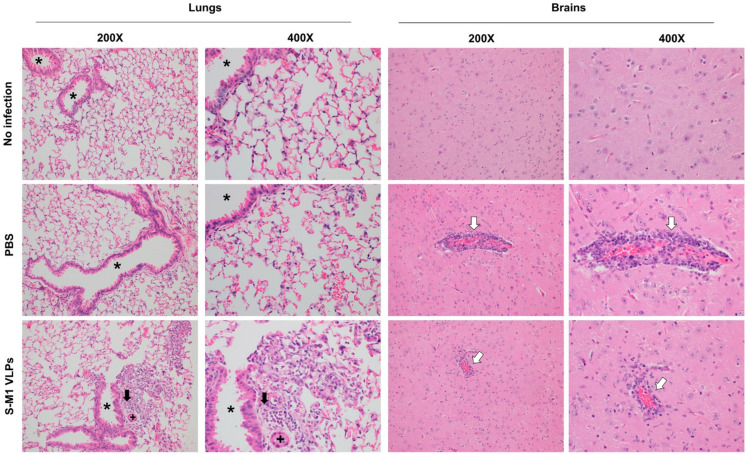
Histopathological analyses of S-M1 VLP vaccinated mice. Tissue samples were collected *post mortem* and embedded in paraffin. The samples were then sectioned and stained with hematoxylin-eosin. * indicates airway, + indicates blood vessel, black arrows indicate peribronchiolar accumulation of lymphocytes, plasma cells, and histiocytes. White arrows indicate Virchow–Robin spaces expanded by lymphocytes and plasma cells.

Histopathological analyses were performed on the tissues of the challenged mice. The lungs of both animals inoculated with PBS and S-M1 VLPs showed mild to marked lymphoplasmacytic and histiocytic pulmonary inflammation. Photomicrographs of the lungs show perivascular and peribronchiolar accumulations of lymphocytes, plasma cells, and histiocytes. Additionally, alveolar macrophages were scattered within the airways (Figure 7). Notably, it is unusual that the PBS group in this study had such a low lung pathology (Figure 7). The clinical observations, ISH results, and laboratory findings correlate with an adequate response to a challenge/disease, but the lung pathology is minimal or even normal in some mice. However, there was mild to marked lymphoplasmacytic and histiocytic pneumonia in only two of the inoculated animals. Overall, the lesions are typically regionally variable. Within the central nervous system, the lymphoplasmacytic and histiocytic inflammation were noted in both the PBS and S-M1 VLPs groups, resulting in the expansion of the vascular walls within the multifocal vessels in the cerebrum (Figure 7). The vasculature was obscured by significant numbers of histiocytes, lymphocytes, and plasma cells, as well as rare neutrophils which largely expanded the Virchow–Robin space and infiltrated the adjacent parenchyma. There was multifocal gliosis, rare satellitosis, and multifocal parenchymal edema in areas adjacent to the inflammation, suggesting a robust inflammatory response leading to encephalitis.

**Figure 8:**
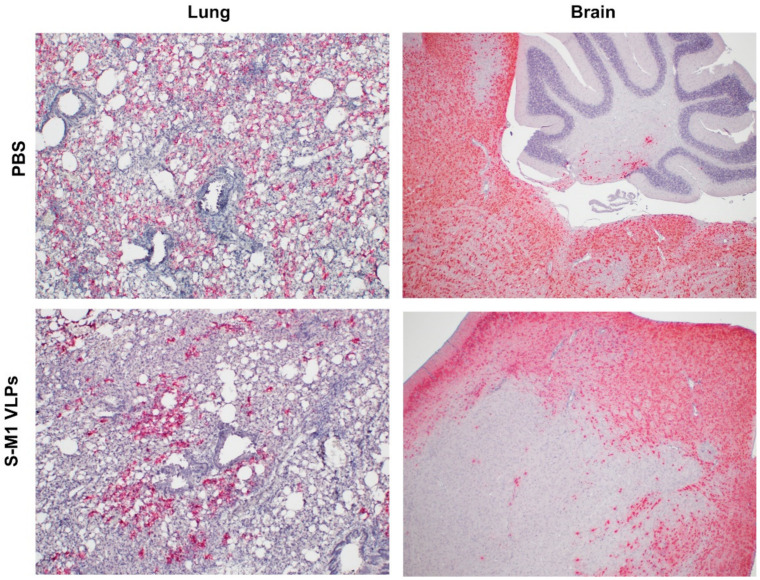
In situ hybridization for SARS-CoV-2 RNA in lung and brain samples. Tissue samples were collected *post mortem*. Formalin-fixed paraffin-embedded (FFPE) tissues of lungs and brain were performed by using the RNAscope^®^ in situ hybridization (ISH) assay. The V-nCoV2019-S RNAscope^®^ probe used is specific for the SARS-CoV-2 Wuhan-Hu-1 S gene (Genbank accession number NC_045512.2, targeting region 21631-23303).

In situ hybridization (ISH) revealed greater amounts of SARS-CoV-2 RNA in the lungs of the PBS group compared to those of the S-M1 VLP group (Figure 8), in agreement with the viral RNA copies quantified by RT-qPCR (Figure 5E). However, the difference between the two groups was not substantial. Brain tissue from all mice in both the PBS and S-M1 VLP groups was moderately to strongly positive for viral RNA in all tissue sections, including the cerebellum, brainstem, hippocampus, thalamus, and cerebral cortex. The cerebellum appeared to be the least affected when included in the section. Brain images in Figure 8 show positive neurons in the brainstem, with minimally positive staining in the cerebellum, of a mouse from the PBS group and regionally variable positive staining in the cerebrum of a mouse treated with an S-M1 VLP. Staining was restricted to the neuronal cytoplasm and dendrites in all sections. It was often regionally variable and affected the majority of neurons in certain areas, whereas others were predominantly negative. Brain ISH scores between groups were not significantly different (PBS: 4.167 ± 0.1667; S-M1 VLPs: 3.667 ± 0.2108).

Overall, histopathological and ISH analyses indicate that, contrary to our expectations that the S-M1 VLP-mediated suppression of viremia would result in a less systemic spread, virus-associated inflammation and viral RNA were observed in both the control and vaccinated animals. With half of the vaccinated mice meeting humane endpoints before the end of the experimental period, compared to all the PBS-treated mice, these results suggest that the S-M1 VLP vaccination may help reduce disease severity to a certain extent.

## 4. Discussion

In this study, we successfully developed a cell line that stably and continuously generates chimeric VLPs based on the influenza virus M1 protein and SARS-CoV-2 S fused with the influenza virus HA transmembrane domain and the cytoplasmic tail. This approach was able to generate VLPs containing the S on their surface in their uncleaved form. When immunized in mice, these chimeric VLPs elicited robust humoral immune responses that were able to neutralize the ancestral strain of SARS-CoV-2 and maintain varying degrees of neutralizing activity against the alpha, beta, gamma, and delta variants, but not against omicron. Cellular immunity was also induced, along with balanced Th1/Th2 responses. While there was a slight trend toward viral load reduction in the lower respiratory tract, the effect of vaccination on the viremia suppression was considerable. The vaccine also improved the survival of mice after a SARS-CoV-2 challenge. However, the histological findings indicated that the S-M1 VLPs elicited immune responses that only partially protected the mice from severe disease, but not from viral invasion of the brain and other organs.

Recently, an influenza virus-based SARS-CoV-2 VLP construct was reported, with the vaccine produced in SF9 cells by the co-transfection of baculovirus plasmids expressing S and M1 proteins. In this study, mice receiving the VLPs elicited neutralizing antibodies; however, the vaccine efficacy was not evaluated [22]. While we have also used influenza virus M1 as the structural basis for VLP self-assembly, our development of an S-M1-expressing cell line that constitutively releases VLPs into the cell culture supernatant will greatly facilitate cost-effective and large-scale VLP production. It has been shown that target antigens must be present on the surface of VLPs in a high density to induce a high-titer antibody response. As shown by α-spike immunogold labeling (Figure 3B), the membrane-associated SARS-CoV-2 S protein was densely localized on the S-M1 VLP membrane. As shown in Figure 4A,B, mice immunized with chimeric S-M1 VLPs elicited strong anti-S antibody responses. In addition, from the presence of IFN-γ-secreting S-specific T cells, our S-M1 VLPs could elicit robust cell-mediated immune responses. While previous studies reported that VLP epitopes could be presented to dendritic cells by both MHC I and II [13], leading to the activation of helper and cytotoxic T cells, whether S-M1 VLPs can elicit antigen-specific cytotoxic T lymphocyte (CTL) responses remains to be tested. Furthermore, although many VLPs do not require co-administration of an adjuvant due to their inherent property of displaying multimeric antigens, the addition of alum to our S-M1 VLP increased its efficacy and enhanced its adaptive immune responses.

The influenza M1 protein, via interactions with lipid membranes, cytoplasmic tails of transmembrane glycoproteins, and M1–M1 interactions, helps mediate virus budding from the cell surface [30]. The M1 anchoring of the HAcyt-fused SARS-CoV-2 S likely provided a three-dimensional architecture similar to the native SARS-CoV-2 S trimer, thereby promoting a strong neutralizing antibody in the vaccinated mice. The use of other viral proteins, such as the VLP base, may organize the trimeric S in different distributions and densities, and allow modulation of the immune response against the S protein.

Both humoral immunoglobulin (IgA and IgG) and T cell responses were demonstrated to contribute to protective immune responses against the SARS-CoV-2 infection [31,32]. While sterilizing immunity against SARS-CoV-2 appears to be mediated mainly by neutralizing antibodies, cell-mediated immunity, particularly CTL responses, may play a crucial role in reducing the severity of symptoms, in the elimination of infected cells, and in long-term memory. Although intramuscular administration of S-M1 VLPs elicited a strong neutralizing antibody, the vaccine-induced T cell response needed to be improved to determine how long the immune responses would last.

Despite having high serum-neutralizing antibody titers, mice immunized with S-M1 VLPs were just as susceptible to the SARS-CoV-2 infection via the respiratory mucosa as their control counterparts. Compared to the control mice, all vaccinated mice displayed only mild to moderate clinical symptoms, lower viral titers in the lungs, and, most importantly, an undetectable virus in the bloodstream. Despite complete viremia suppression, significant lesions were detected in multiple body systems, indicating a systemic spread. Furthermore, due to the intramuscular administration, our vaccine did not produce detectable S-specific mucosal secretory IgA antibodies. We believe that novel VLP formulations that allow mucosal administration, particularly via the upper respiratory tract, could improve the S-M1 VLP-based vaccine’s protective efficacy against SARS-CoV-2.

Currently, many technical and practical challenges associated with large-scale production limit the use of VLPs to small-scale research, although their immunogenicity has been demonstrated in many preclinical studies. In particular, VLPs that are structurally composed of multiple protein components encounter difficulties in upscaling to obtain highly purified VLPs. Combinations of different purification methods such as size exclusion chromatography and ion exchange chromatography could be used. However, these methods would inevitably increase the complexity and cost of S-M1 VLP production. Factors such as storage temperature, pH, and stabilizer/buffer could also affect the integrity and stability of VLP particles, thus determining the efficacy of S-M1 VLPs. While we have attempted to take advantage of the HEK293T cell line that constitutively expresses both SARS-CoV-2 S and influenza M1 proteins, this mammalian cell-based S-M1 VLP production system requires further process optimization to achieve a high purity and yield which is suitable for large-scale production.

Unlike many subunit vaccines, which generally lack the native danger signals that are critical for stimulating immune responses, VLPs possess not only inherent multimeric structures, but also various pathogen-associated molecular patterns (PAMPs) that resemble wild-type viruses. By combining many surface PAMPs with highly repetitive epitopes on the particles, VLPs have unique features that confer superior immunogenic properties. While these unique properties make VLPs a potential COVID-19 vaccine platform from an immunological perspective, much work remains to be done to translate this immunogenicity into robust and long-lasting protection.

## Figures and Tables

**Table 1:** Clinical signs observation.

Group	Day Post-SARS-CoV-2 Challenge						
	0	1	2	3	4	5	6
PBS	Normal 6/6	Normal 6/6	Normal 6/6	Normal 6/6	Normal 5/6 Lethargy and ocular discharge 1/6	Anorexia 5/6 Increased respiration 5/6 Greatly labored respiration 1/6 Moved slowly after moderate stimulation 6/6 Mildly rough hair coat 6/6	-
S-M1 VLPs	Normal 6/6	Normal 6/6	Normal 6/6	Normal 6/6	Normal 6/6	Anorexia 3/6 Increased respiration 2/6 Lethargy 1/6 Moved after mild stimulation 2/6 Moved slowly after moderate stimulation 1/6 Mildly rough hair coat 3/6	Lethargy 3/3 Mildly rough hair coat 3/3

## Data Availability

The data presented in this study are available in the article.

## Disclaimer

Material has been reviewed by the Walter Reed Army Institute of Research. There is no objection to its presentation and/or publication. The opinions or assertions contained herein are the private views of the author, and are not to be construed as official, or as reflecting true views of the Department of the Army or the Department of Defense. The research was conducted under an approved animal use protocol in an AAALAC accredited facility in compliance with the Animal Welfare Act and other federal statutes and regulations relating to animals and experiments involving animals, and adheres to principles stated in the Guide for the Care and Use of Laboratory Animals, NRC Publication, 2011 edition.
